# Research on elderly users' intentions to accept wearable devices based on the improved UTAUT model

**DOI:** 10.3389/fpubh.2022.1035398

**Published:** 2023-01-09

**Authors:** Junxun Chen, Tao Wang, Zhenyu Fang, Hongtao Wang

**Affiliations:** ^1^School of Economics and Management, China Jiliang University, Hangzhou, China; ^2^School of Management, Shanghai University, Shanghai, China

**Keywords:** elderly users, wearable devices, improved UTAUT, intention to use, technology acceptance

## Abstract

**Introduction:**

As the proportion of the world's elderly population continues to increase, wearable devices can provide ideas for solving a series of problems caused by population aging. Therefore, it is of great significance for the development of intelligent elderly care and the improvement of the quality of elderly care services to explore the factors that influence the intention of elderly users to accept wearable devices.

**Methods:**

An improved unified theory of acceptance and use of technology (UTAUT) model is constructed from the perspective of elderly individuals, and new parameters are added, including four factors related to wearable devices, including performance expectancy, perceived cost, hedonic value and aesthetic appeal, and three factors related to elderly individuals, including personal physiological conditions, health anxiety and personal innovativeness in information technology. The data analysis was accomplished with the partial least square regression structural equation modeling.

**Results:**

The findings of this study revealed that performance expectancy, perceived cost, hedonic value and aesthetic appeal all have significant impact on elderly users' intention to use wearable devices. Furthermore, personal innovativeness in information technology, personal physiological condition, and intention to use all have significant impact on elderly users' actual usage behavior of wearable devices. However, there is no obvious relationship between health anxiety and actual usage behavior.

**Discussion:**

Elderly adults' attention to wearable devices plays an important role in the development of the wearable device-related industry chain, which provides management suggestions for stakeholders.

## 1. Introduction

As the populations of Europe, America, China, Japan, and other countries gradually age, it is very important to solve the problems related to medical care brought about by aging (1). However, the medical care industry for the elderly faces a mismatch between supply and demand (2). This mismatch is mainly manifested in the unbalanced global distribution of medical and health resources, most of which are concentrated in large cities, making it difficult to reach grassroots residents who have greater needs (3, 4). This results in an imbalance in the supply and demand of elderly care services, which has placed huge pressure on elderly individuals (5). Therefore, as a new technology and innovation that can solve the above problems, wearable devices can be used to take care of elderly individuals in their own homes. This approach is economical and can alleviate the increasing social support costs caused by aging (6).

At present, many companies are investing heavily in research on wearable devices, indicating that wearable devices have value for health monitoring and information tracking and can be further used by hospitals and families to improve the health and self-care of patients. Bölen (7) held that wearable devices should be more targeted to specific diseases and specific groups of people. Wu et al. (8) believed that wearable devices are easy to operate and easy to carry and have calling and positioning functions. Therefore, wearable devices are in demand for elderly people who have only recently been exposed to smart products. Wearable devices can build a bridge between doctors and patients to communicate, help patients relieve pain, solve disease problems, and improve work efficiency for doctors with a high prevalence of elderly patients (9).

Wearable devices integrate science, technology, aesthetics, and other elements, but user needs play a crucial role from the perspective of industry development. There are only a few studies on wearable technology for elderly users, but there are still some shortcomings. First, consumers (especially elderly users) have low acceptance of wearable devices (10). Second, wearable devices not being functional enough, being too algorithmically complex, and not being comfortable enough lead to low satisfaction with wearable devices for consumers (11). Last, wearable technology still has a long way to go from the period of commercial exploration to the maturity of large-scale applications (12).

The objectives of this study are as follows: On the one hand, what factors influence the behavioral intention of elderly users to accept and use wearable devices? On the other hand, what theoretical framework is suitable for analyzing the intention of elderly users to accept these devices?

Therefore, this study explores the intention of consumers to use wearable devices from a new perspective of elderly users and finds important factors that affect the use of wearable technology by elderly users. It can help elderly users manage their health, bring more possibilities for solving pension problems, and realize intelligent pension services. On the one hand, this study can help alleviate the burden of medical care brought about by population aging and the pressure on the social health care system and provide security for the elderly in society, especially the empty-nest elderly. On the other hand, wearable devices can quickly and conveniently collect user physiological data, which allows the health of the elderly in the postepidemic era to be effectively managed and effectively reduce the possibility of accidents among elderly individuals.

## 2. Literature review

### 2.1. A review of wearable devices

Regarding the definition of wearable devices, there is currently no unifying statement in Academia, and this research use the form of a list to illustrate the definition of wearable devices proposed by different scholars, as shown in Table 1. With respect to the research of wearable devices, technical issue related to smart wearable devices is the most concerned topic. Most of the studies in the topic unfold around the aspects such as the interface design, functionality and practical quality of smart wearable devices. For example, Matuska et al. (17) designed an entirely new wearable device by modifying the independent accelerators, gyroscopes, and microcontrollers. Juhlin et al. (18) designed a stylish visual screen for wearable devices.

**Table 1:** Research on the definition of wearable devices.

**References**	**Definition**
Liu and Guo (13)	Wearable computers with a mobile Internet connection that are worn like dresses and personal adornments to display information for users intelligently and efficiently, such as wearable glasses and wearable watches.
Yildirim and Ali-Eldin (14)	Electronics or computers that can be worn on the body when inserted into items of clothing and accessories.
Farivar et al. (15)	The devices that will be physically attached to the users in order to monitor some aspects of their behaviors, such as their physical activity (number of steps, distance, calories burned, etc.) and their vital signs (heart rate, blood pressure, etc.).
Wei (16)	A portable device that is worn directly on the body or integrated into the user's clothes or accessories. It is not only a hardware device, but also realizes powerful functions through software support, data interaction, and cloud interaction.

### 2.2. A review of user behavior of wearable devices

Research on wearable devices in the academic field tends to start from certain perspectives, such as sensor type, data extraction and classification methods, but there are relatively few studies on user preferences and behavioral intentions.

For instance, Dehghani et al. (19) exploited an extended technology acceptance theory to verify the driving factors that influence continuous intention and actual use of wearable devices, and modeling using partial least squares paths from the data collected from 383 actual smartwatch users. Kim and Shin (20) discussed users' cognition degree of smart wearable devices and used confirmatory factor analysis and structural equation modeling methods to investigate the determining factors of users' intention to use smart wearable devices. Bölen (7) believed that aesthetics, satisfaction, individual mobility, and habits are essential factors which influence people's acceptance of smart wearable devices, and verified this assumption through structural equation models. Yang et al. (21) explored and demonstrated the intention and adoption of Wearable Fitness Devices among Chinese adults. Hayat et al. (22) investigated that the perceived product value instigates the intention to use wearable medical devices and health motivation, and the intention to use promotes the adoption of wearable medical devices.

With respect to the wearable devices, although some scholars have studied the influencing factors of user preferences and behavioral from the perspective of the theory of planned behavior, the unified theory of acceptance and use of technology, and innovation diffusion theory, existing studies are mostly concentrated on young users (15, 23). At the same time, the current development of the wearable device industry is limited by issues such as user privacy and a lack of featured products (24). According to the survey, most of the consumers are skeptical of products related to wearable devices, with only a few consumers willing to accept them (21).

### 2.3. Theoretical reviews

The theory of reasoned action (TRA), the theory of planned behavior (TPB), the technology acceptance model (TAM), and other theories provide a theoretical basis to continuously enrich and develop research on users' intentions to use information technology. These theoretical models are often used to study individual adoption behaviors, information platform usage, and information service usage. They have strong applicability, but the influencing factors are too general and lack consideration of individual differences and contextual factors (25–27). The two important factors in the technology acceptance model are perceived usefulness and perceived ease of use, which are used to explain the factors that affect the use of information systems. The model cannot fully represent the relationship between the variable factors, nor can it find all the factors that hinder the acceptance of the technology. Therefore, these models have certain limitations.

Based on the above model, Venkatesh et al. (28) proposed the unified theory of acceptance and use of technology (UTAUT). This model is an integration of the TRA, TPB, TAM, and other models and can better reflect the influence of individual users' knowledge, experience, and intentions in accepting new information technology. It has been widely verified that the explanatory ability of users' intentions to accept reaches 70%.

## 3. Conceptual model and hypothesis development

### 3.1. Conceptual model

UTAUT, which has yielded rich research results and a wide range of applications, includes four core variables that affect intention to use and use behavior, namely, performance expectancy, effort expectancy, social influence and facilitating conditions. However, judging from the current research progress, the impact of these four core variables on users' intentions to use varies in degree across industries and scenarios (29). Therefore, the application of the UTAUT model needs to be combined with the environmental characteristics to obtain a targeted rule of user intention.

**Figure 1:**
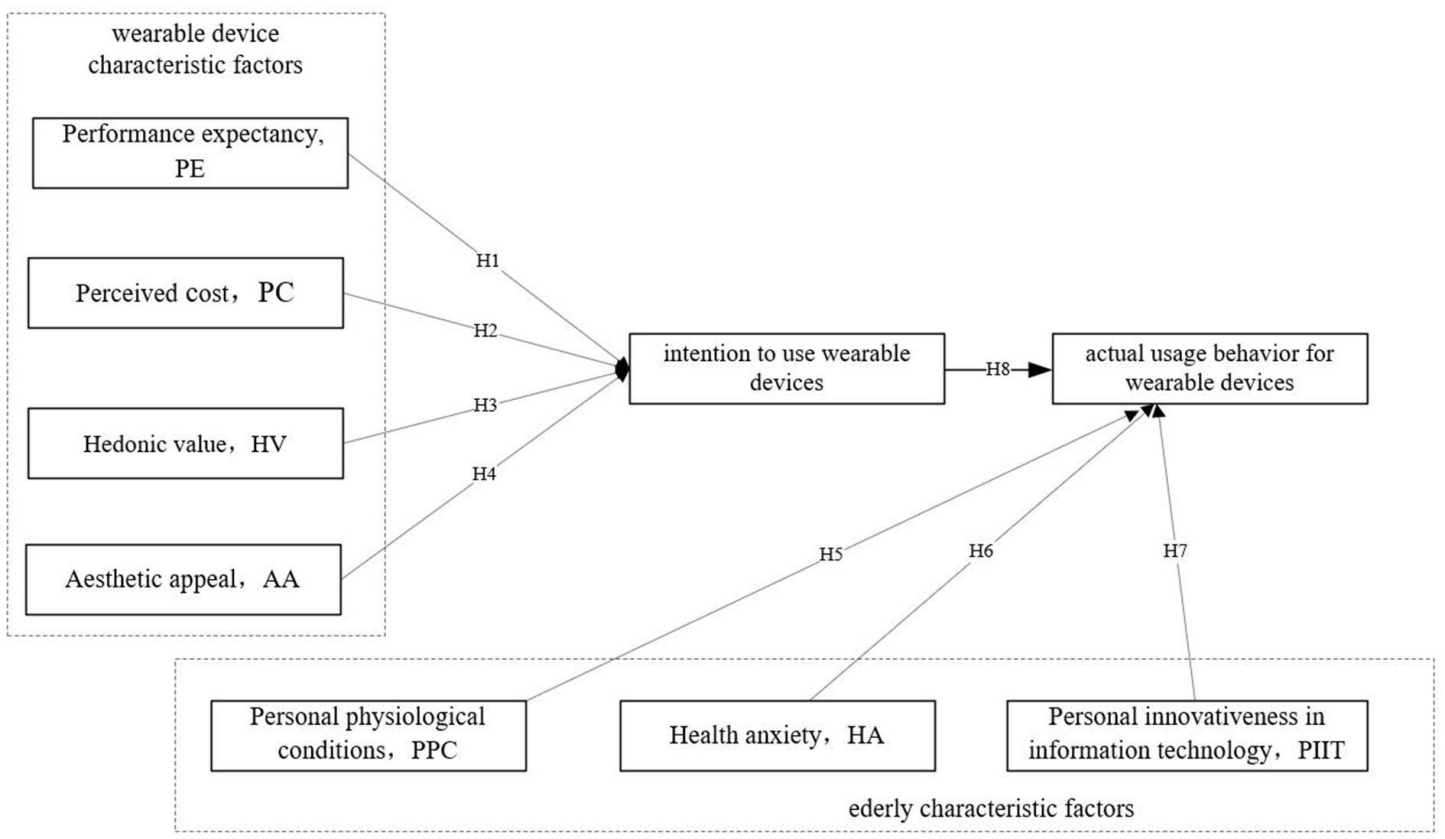
Proposed improved UTAUT in the present study.

Considering that the subjects studied in this article are elderly people, due to a minute difference caused by age, and according to the description of Bu et al. (30), factors such as gender and experience cannot be well applied as moderating variable data for elderly people. As a result, this study deletes the moderation variable data in the basic UTAUT model. At the same time, combined with the characteristics of wearable devices, the original convenience factors and social influences are replaced with factors that can better reflect their characteristics. Overall, this study designs a modified UTAUT model and combines the characteristics of wearable devices to better explore the transmission mechanism of elderly users' purchase intentions for wearable devices (see Table 1). The construction model in this paper includes two dimensions, the elderly characteristic factors and the wearable device characteristic factors, involving 6 variable data. The characteristic factors of wearable devices include performance expectations, perceived cost, hedonic value, and aesthetic attraction, while the characteristic factors of the elderly are health anxiety, personal innovativeness in information technology, intention to accept, and actual use behavior. This specific structure is shown in Figure 1.

This study theoretically intends to solve the following research questions according to the above study.

1. What functions of wearable devices can encourage the elderly to adopt wearable devices?
2. Do the physical health situations and psychological factors of the elderly affect the use of wearable devices?

### 3.2. Development of hypotheses

#### 3.2.1. Hypothesis related to wearable device characteristic factors

##### 3.2.1.1. Performance expectancy (PE)

In the UTAUT model, performance expectancy is defined as “the degree to which an individual feels that using the system is helpful to his or her job” (31). Performance expectancy is a major determinant of behavioral intent to use technology. In the context of wearable devices, users are more likely to adopt technology when they believe that wearable devices enable them to improve healthcare efficiency (30). Therefore, this paper defines performance expectancy as “users' perceptions of the usefulness, extrinsic motivation, and outcome expectancy of wearable devices” and proposes the following hypothesis:

H1: PE has a significant positive impact on elderly users' intentions to use wearable devices.

##### 3.2.1.2. Perceived cost (PC)

Economic issues have been identified as a major obstacle in the dissemination of information technology and services. Perceived cost is the tendency of users to compare the potential benefits and consumption costs of technologies and services (32). Based on the definition of perceived cost in several previous studies (33), this study defines perceived cost as “the burden of costs consumed by purchasing, using, and maintaining wearable devices”. In the context of wearable devices, the relationship between perceived cost and satisfaction with using the device was confirmed. For example, Dehghani et al. (19) argue that consumers' intentions to purchase specific products and services are negatively affected by their perceived cost in mobile marketing services. Therefore, based on the above discussion, the following hypothesis has been proposed:

H2: PC has a significant negative impact on elderly users' intentions to use wearable devices.

##### 3.2.1.3. Hedonic value (HV)

Some studies on information systems and user experience suggest that the hedonic values users assign information systems and services can be viewed as a key motivation for their perceptions of systems and services. Yang and Lee (33) define HV as “the degree to which the use of a particular technology or service is regarded as an enjoyment”. Building on this definition, the current study defines HV as “the degree to which the use of wearable devices is perceived as enjoyment”. Spreer and Rauschnabel (34) found a close relationship between users' perceived satisfaction and enjoyment when explaining their experience on a mobile video app for 270 responses. Therefore, based on the above discussion, the following hypothesis has been proposed:

H3: HV has a significant positive impact on elderly users' intentions to use wearable devices.

##### 3.2.1.4. Aesthetic appeal (AA)

Aesthetic appeal is the level of feeling associated with style or fashion (35). Previous research has pointed out that aesthetic appeal is an important factor in making product consumption decisions. Sonderegger and Sauer (36) investigated how variables such as perceived visual appeal affect the usability of smartphones. Their results show that wearable devices with perceived visual appeal have overall better perceived performance than visually unappealing smartphones. Wearable devices are in the early stages of adoption, and aesthetic and visual appeal can be an important basis for consumer decision-making (37). According to the research of Jeeyeon et al. (38), wearable devices as a fashion product, their exquisite appearance and their unique design structure increase users' intentions to adopt. Therefore, based on the above discussion, the following hypothesis has been proposed:

H4: AA has a significant positive impact on elderly users' intentions to use wearable devices.

#### 3.2.2. Hypothesis related to the elderly characteristic factors

Jain et al. (39) found evidence of an association between satisfaction and adoption intention through the UTAUT model. From the perspective of consumers, the acceptance intentions of elderly people is a key driver of their actual use behaviors, and it is also considered to be the premise of their repurchase intentions. However, elderly users have corresponding behaviors and attitudes based on their own special reasons and then make adoption decisions. Hypothesis are proposed as following.

##### 3.2.2.1. Personal physiological conditions (PPC)

Personal physiological conditions include hearing, vision, speech, and memory impairments, as well as a range of challenges to device use. Because of the decline in hearing, vision, speech and cognitive abilities associated with aging, elderly people have difficulty concentrating on complex problems with hardware and software devices. As an instrument to assist in monitoring physical health, wearable devices can provide significant information such as blood pressure, blood sugar, heartbeat, and temperature. Edelstein et al. (40) argued that the underlying diseases induced by personal physiological conditions cause elderly users to have more trouble using wearable devices, which makes them more likely to go to the hospital or be cared for by their children. However, elderly individuals who are in good physical condition may spend more time on exercise and pay attention to the monitoring of blood pressure, blood sugar, and other indicators to prevent various diseases (41). Therefore, PPC can act as an internal control or inhibitory condition that affects the use of wearable devices by elderly users. Thus,

H5: PPC has a significant positive impact on elderly users' intentions to use wearable devices.

##### 3.2.2.2. Health anxiety (HA)

HA is individuals' concern or fear about their own illness or potential future health symptoms. Thatcher et al. (42) defined health anxiety as individuals' worry about their future health when they do not have a disease or excessive worry about health problems when they do have a disease. Cabrera et al. (43) hold that individuals with health anxiety tend to adopt safe behaviors, which may be aimed at reducing health-related fears. Therefore, this paper claims that elderly individuals with health anxiety may obtain their own body-related index parameters from wearable devices to reassure themselves. This result also shows that elderly individuals with high health anxiety utilize wearable devices as a medical monitoring function to a greater extent than elderly individuals with low health anxiety. Thus,

H6: HA has a significant positive impact on elderly users' intentions to use wearable devices.

##### 3.2.2.3. Personal innovativeness in information technology (PIIT)

PIIT is the intention of individuals to try new information technology. Agarwal and Prasad (44) defined PIIT as personal characteristics that are relatively stable in individuals and remain unchanged across contexts. Kurata (45) pointed out that PIIT is generally not affected by environmental or internal variables. The role of this trait remains stable in specific configurations of individual and situational factors. Therefore, this paper argues that personal innovativeness in information technology is an inherent attribute of individuals with an adventurous spirit. This risk-taking tendency encourages individuals to seek out new and innovative experiences. Obviously, elderly individuals with higher PIIT will be more willing to accept wearable devices and try new things. Thus,

H7: PIIT has a significant positive impact on elderly users' intentions to use wearable devices.

##### 3.2.2.4. Relationship between intention to use (IU) and actual usage behavior behavior (AUB)

Many theories related to technology acceptance, such as TAM and UTAUT, verify the relationship between use intention and user behavior. Many scholars have also verified the relationship above. For example, Hanif and Lallie (46) used a modified UTAUT model to measure the intention of British citizen aged over 55 to use mobile banking applications, hypothesizing that the intention to use positively impacts actual use behavior. Thus,

H8: Elderly users' intentions for wearable devices has a significant positive impact on their actual usage behavior for wearable devices.

## 4. Research methodology

### 4.1. Research design

The applicability of the questionnaires included in this study was ensured, and they were carefully selected from published information. To ensure the feasibility of the questionnaire, a pretest was conducted in some elderly participants who wore wearable devices. Both statements were slightly modified based on the feedback received from the elderly individuals who had completed the pretest to improve their understanding of the variable data. The questionnaire contains three sections. The first section is the classification of the questions, which is used to filter out those users who have used or are familiar with wearable devices. The second section is demographic information, such as gender and living conditions. The third section is a rating scale question, using a 5-point Likert scale (1 = “strongly disagree”; 5 = “strongly agree”) to measure each variable in the study. The relevant measurement items are shown in Table 1. Table 2 shows the demographic characteristics of the respondents.

**Table 2:** Items and contents of measurement constructs.

**Constructs and sources**	**Items and contents**
PE (31)	PE1: I think wearable devices are beneficial.
	PE2: I think using wearable devices improves my life.
	PE3: I think using wearable devices brings more convenience.
PC (19)	PC1: I think there are financial barriers t using wearable devices.
	PC2: I think wearable devices are expensive.
	PC3: Overall, it cost me a lot of money to use wearable devices.
HV (36)	HV1: Interacting with wearable devices is fun.
	HV2: I like using wearable devices.
	HV3: Using wearable devices gives me a lot of enjoyment.
AA (19)	AA1: I find wearable devices to look attractive.
	AA2: I think wearable devices are beautiful.
	AA3: I think the design of wearable devices is professional.
PIIT (44)	PIIT1: I am willing to use new information technology.
	PIIT2: I think it is fun to try new information technology.
	PIIT3: I enjoy trying new information technology.
PPC (47)	PPC1: My physical condition makes my daily activities strenuous.
	PPC2: My physical condition limits the kinds of activities I can do.
	PPC3: My physical condition makes my daily activities difficult.
HA (48)	HA1: I am worried that I have a serious illness.
	HA2: I am worried about my health.
	HA3: If I hear about certain disease, I think I have it myself.
	HA4: I usually feel at risk of serious illness.
IU (49)	IU1: I would like to use wearable devices.
	IU2: I will recommend wearable devices to others.
	IU3: Related companies should vigorously develop wearable devices.
AUB (50)	AUB1: I use wearable devices a lot.
	AUB2: I will share wearable devices with others.
	AUB3: I will continue to use wearable devices.

### 4.2. Sampling strategy and sample collection

The sample collection strategy of the study was divided into the following three stages.

In the first stage, interviews were used to obtain qualitative data. The interviews were conducted in an elderly welfare center in Hangzhou, Zhejiang Province. The elderly welfare center provided opportunities for the elderly to continue learning and improve their quality of life. The respondents were all over 60 years old. The main questions of the interview focused on their current activity levels, health conditions, whether they had used wearable devices and their views on wearable devices. Considering that some elderly individuals may not know about wearable devices, we provided 40 Fitbits for the elderly participants.

In the second stage (1 week after the first interview), the main task was to ask the elderly to fill in the survey questions to obtain their views on wearable devices. A total of 40 questionnaires from the elderly welfare center were used as predictive test samples to test the rationality of the questionnaire, and the questionnaire was finally modified and improved according to the feedback results of the pretest samples. After improving the details of the questionnaire distributed in the field survey, a formal questionnaire was obtained again. This approach can improve the efficiency and quality of the questionnaire when it is officially distributed, reduce unqualified questionnaires, and improve the practicality and scientificity of the questionnaire.

In the third stage, expanded investigation, the survey scope was expanded from the elderly activity center in Hangzhou to other places. For example, in shopping malls, elderly gathering centers and other places.

This paper used the following methods to reduce the impact of sampling error. First, in terms of sampling error, cross-checking was used by multiple members to reduce entry errors and ensure data accuracy, which control the errors caused by mistakes in registration. Second, this paper strictly abode by the principle of random. In the process of selecting data, the sample indicators with too high or low values were excluded which can reduce the systematic representative error. Third, to avoid the invalid answer caused by respondents which were unable or unwilling to cooperate, the questionnaires were designed to be more concise and clear, and the length of each items were controlled to reduce impatiently perfunctory answer. At the same time, each steps of the procedure were carefully arranged to prevent various loopholes and reduce the rejection rate of the respondents. Fourth, before distributing the questionnaire, survey members were carried out in centralized training for understand the questionnaire design as well as the skills of communicating with the respondents. For example, for some elderly people with poor hearing and presbyopia, survey members need to dictate each question on the questionnaire and explain the meaning of the questions and options in detail. When the elderly are unable to make a judgment, survey members will ask them carefully to help them make a judgment. Finally, all the survey members were organized with a strict standard. An intensive training was held for all the survey members before the questionnaire to ensure that they are familiar with all the questions on the questionnaire. Inductive words were prohibited in the process of survey. In addition, the workload assigned to each survey member was reasonable enough in order to avoid increasing measurement errors due to excessive survey intensity.

When determining the sample size, we weighed from time and the economical aspects. In order to make the survey results more scientific and representative, it is hoped that the maximum absolute error of the survey results lower than = 5%, and the significance level is 0.05, the confidence level Z_α/2_ is 1.96. The initial sample size was calculated when it reached the maximum value (i.e., *p* = 0.5). The estimate of the sample *n*_1_ size can be taken:


$$\begin{array}{l} \;\Delta =0.05,\alpha =0.05,Z_{\frac{\alpha }{2}}=1.96,p=0.5\\ n_{1}\geq \frac{Z_{\frac{\alpha }{2}}{}^{2}\times p(1-p)}{\Delta^{2}}=\frac{1.96^{2}\times 0.5(1-0.5)}{0.05^{2}}=384.16 \end{array}$$

Furthermore, considering that the estimated response rate is 96%, the final sample size is *n*_2_:


$$\begin{array}{l} n_{2}=\frac{n_{1}}{r}=\frac{384.16}{0.96}=400.16\approx 400 \end{array}$$

A total of 400 questionnaires were sent out this time, and 388 valid questionnaires were collected. Among them, 31 respondents were replaced by the children of elderly individuals, so 357 effective questionnaires were actually collected.

### 4.3. Estimation techniques

The Partial Least Squares (PLS) method, a statistical analysis technique based on the Structural Equation Modeling (SEM), was used to test and validate the proposed model and the relationships among the hypothesized constructs. Hair et al. (51) discussed how the PLS-SEM approach is appropriate for evaluating non-normal data, as are examined here. It is also suggested that PLS-SEM should be applied when developing an initial theory for exploratory research models. So, Smart-PLS software, one of the well-known software applications for PLS-SEM, was used to analyze the data (22).

## 5. Analysis and results

### 5.1. The measurement model assessment

Research models were explored using confirmatory factor analysis and SEM methods. Guidelines from previous studies required Cronbach's α over 0.7, factor loadings over 0.7, and AVE over 0.5 (52, 53). If VIF value is less than 5 (strictly 3), it indicates that the model has no multicollinearity problem and the model is well-constructed. On the contrary, if VIF is greater than 5, the model construction is poor (54).

Table 3 shows that all the Cronbach's α of this study are greater than 0.8, and CRs are greater than 0.8 which indicates that this model has a good reliability. We also found that the VIFs were approximately 1.819 with a maximum of 2.408. The values were less than the critical threshold of five and far less than the conservative threshold of 10.

**Table 3:** Standardized factor loadings, VIFs, CRs and AVEs, Cronbach's α in this study.

	**Factor loadings**	**VIF**	**Cronbach's α**	**CR**	**AVE**
PE1	0.843	1.977	0.846	0.903	0.762
PE2	0.913	2.127			
PE3	0.861	2.018			
PC1	0.877	1.657	0.782	0.955	0.667
PC2	0.689	1.651			
PC3	0.870	1.577			
HV1	0.655	1.818	0.808	0.770	0.568
HV2	0.554	1.925			
HV3	0.985	1.620			
AA1	0.896	1.526	0.758	0.849	0.660
AA2	0.799	2.350			
AA3	0.848	2.408			
PIIT1	0.896	1.817	0.826	0.885	0.720
PIIT2	0.799	1.968			
PIIT3	0.848	1.812			
PPC1	0.752	1.963	0.821	0.865	0.684
PPC2	0.738	1.593			
PPC3	0.971	2.058			
HA1	0.893	2.163	0.837	0.882	0.653
HA2	0.780	1.250			
HA3	0.711	2.054			
HA4	0.837	2.146			
IU1	0.847	1.331	0.902	0.770	0.547
IU2	0.833	1.468			
IU3	0.810	1.171			
AUB1	0.923	2.061	0.828	0.883	0.654
AUB2	0.779	1.721			
AUB3	0.428	1.702			

Discriminant validity was calculated according to Heterotrait-Monotrait Ratio (HTMT) by Henseler et al. (55). The discriminant validity has been established between two reflective constructs if the HTMT value should be below 0.9. According to Table 4, the HTMT criterion is considered to be satisfied since all the values were below the suggested value of 0.9. Therefore, the discriminant validity is established.

**Table 4:** Discriminant validity (HTMT criterion).

**Construct**	**PE**	**PC**	**HA**	**AA**	**PIIT**	**PPC**	**HA**	**IU**	**AUB**
PE									
PC	0.661								
HV	0.611	0.645							
AA	0.754	0.759	0.710						
PIIT	0.544	0.619	0.506	0.637					
PPC	0.832	0.757	0.645	0.804	0.675				
HA	0.807	0.738	0.704	0.796	0.664	0.845			
IU	0.072	0.282	0.230	0.248	0.234	0.103	0.148		
AUB	0.150	0.342	0.126	0.158	0.115	0.179	0.232	0.187	

### 5.2. The structural model assessment

To assess the proposed hypothesis, the regression analysis is applied in Table 5. In the PLS-SEM, the *R*^2^-value is applied to assess the model's explanatory power. The target is to have a high *R*^2^-value to explain the endogenous latent variance. *R*^2^-values range from 0 to 1. The higher the value, the better is the explanatory power of the model (56).

**Table 5:** Path analysis results.

**Path**	**Standardized coefficient**	***p*-value**	***t*-value**	***R* ^2^**	***Q* ^2^**	***f* ^2^**	**Results**
H1: PE → IU	0.036	0.000	6.138	0.646	0.535	0.167	Supported
H2: PC → IU	−0.623	0.000	2.342			0.008	Supported
H3: HV → IU	0.269	0.001	8.132			0.174	Supported
H4: AA → IU	0.301	0.000	7.289			0.093	Supported
H5: PIIT → AUB	0.424	0.013	3.539	0.492	0.423	0.081	Supported
H6: PPC → AUB	0.081	0.026	10.232			0.053	Supported
H7: HA → AUB	0.069	0.478	1.208			0.000	Not supported
H8: IU → AUB	0.120	0.033	1.353			0.143	Supported

We performed the blindfolding test to get the value of *Q*^2^-value. According to Hair et al. (51), *Q*^2^-values between 0 and 0.25 indicate low out-of-sample predictive power, those between 0.25 and 0.5 indicate moderate predictive power, and those >0.5 indicate good predictive power.

Table 5 shows the *R*^2^ for IU is 0.646, for AUB is 0.492. Table 5 also demonstrates that IU have a high predictive value, AUB have a medium predictive strength.

Furthermore, the effect size, referred to as an *f*^2^, is ranked as small, medium and large. Values above 0.02 and up to 0.15 are considered small; values of 0.15 and up to 0.35 are medium; and values 0.35 and above are large effects (56). In Table 5, the researchers also explained the *f*^2^ and the effect size.

The path analysis results are summarized in Table 5. The factors related to wearable devices—PE (H1, β = 0.036, *t* = 6.138, *p* < 0.001), HV (H3, β = 0.269, *t* = 8.132, *p* = 0.01), and AA (H4, β = 0.301, *t* = 7.289, *p* < 0.001)—positively affect the intentions of elderly users, while PC negatively affects the intentions of elderly users (H2, β = −0.623, *t* = 2.342, *p* < 0.01).

With respect to factors related to the characteristics of elderly individuals, PIIT (H5, β = 0.424, *t* = 3.539, *p* = 0.013) and PPC (H6, β = 0.081, *t* = 10.232, *p* = 0.026) positively affect elderly users' actual behaviors when using wearable devices, but HA has little effect on actual behaviors.

In addition, IU (H8, β = 0.120, *t* = 1.353, *p* = −0.033) also shows a positive relationship with the actual behavior.

## 6. Conclusion and implications

### 6.1. Conclusion

This paper constructed a model to study the factors of the behavioral intentions and actual behaviors of elderly users toward wearable devices. Research hypotheses based on the modified UTAUT model were proposed, and the relevant hypotheses were analyzed and tested by combining questionnaire data and SEM. The final results supported most of the hypotheses except for one. The specific analysis is as follows.

This study found that PE has a significant positive effect on the intention of elderly people to use wearable devices, which is consistent with the results obtained in previous studies (57) and can be explained by the following fact: in environments in which wearable devices greatly improve the efficiency of elderly people's lives, the intention to use wearable devices is more obvious (58). Therefore, wearable device manufacturers should focus on developing wearable device hardware that enhances the expectations of elderly individuals.

This study found that PC has a significant negative effect on the intentions of elderly people to use wearable devices. This is despite many previous studies having identified the perceived cost of wearable devices as the most significant factor affecting the intention to accept (59). This may be explained by the fact that elderly people are more concerned about whether wearable devices can provide them with various convenient uses, such as body monitoring, so they tend to prefer lower-cost wearable devices to reduce the cost of medical treatment (57). Therefore, manufacturers of wearable devices should provide a more convenient functional design aimed at the needs and physiological weight of the elderly and remove some cumbersome and uncommon functions to reduce the production cost.

This study found that HV has a significant positive effect on the intention of elderly adults to use wearable devices, which can be explained by previous work on the relationship between hedonic value and consumer postconsumer feelings (60). Our findings are consistent with those of previous studies because the higher the hedonic value felt by elderly adults when using wearable devices (61), the more positive emotions are generated, thus promoting their wearable device use behaviors.

This study found that AA has a significant positive effect on the intentions of elderly adults to use wearable devices, and Yoon and Cho (62) stated that products that meet or exceed the hedonic needs of users should enhance their sense of pleasure. This is because the stronger the visual aesthetics of a wearable device are, the more attractive it will be to the user, thus increasing the intention to use wearable devices (63).

This study found that PPC has a significant negative effect on the behavioral intentions of elderly adults to use wearable devices, because elderly adults who are in better health don't need it (64). Therefore, it is necessary to identify the target users of wearable devices and highlight common features for healthy populations. The study recommends that manufacturers demonstrate the reliability of the measurement of physical signs for elderly adults and develop products that meet the industry standards of wearable devices (40).

This study found that PIIT had a significant positive effect on the behavioral intentions of elderly adults to use wearable devices because elderly adults who possess a sense of innovation and self-directed learning toward new technologies generate a sense of pleasure when using wearable devices (65). This result was confirmed by Shetu et al. (66). They found that people with high PIIT levels tend to develop more positive attitudes toward new information technology and use it more quickly.

This study found that HA has a non-significant effect on the behavioral intentions of elderly adults to use wearable devices. Elderly users with health anxiety are willing to spend more time and effort finding all devices and tools that can assist in improving their health (67). Therefore, they will not only focus on whether the services provided by wearable devices are beneficial to their health but blame an external factor and seek other medical aids to escape from their current health problems and insecurities (68). With the continuous development of information and the popularization of smartphones, elderly users are expected to welcome wearable device services and attach more importance to a series of benefits brought by wearable devices (69).

### 6.2. Contribution

Elderly adults' attention to wearable devices plays an important role in the development of the wearable device-related industry chain, so this study helps expand the scope of research on wearable devices.

In this study, we improved the original UTAUT model and constructed corresponding parameter variables from the perspective of wearable device function and elderly characteristics. Therefore, this study provides more value for enriching theories related to technology acceptance.

Finally, the conclusions of this study provide more useful information for manufacturers and service providers in the corresponding industries.

### 6.3. Limitations

On the issue of sample selection, this paper mainly focuses on densely populated elderly activity centers but may ignore some elderly people who live at home for long periods or are located in remote areas due to poor health. Therefore, these groups should also be included in subsequent samples.

In addition, there are many kinds of wearable devices, and they are constantly being updated; however, this article investigated only the use of the Fitbit smart bracelet by elderly individuals (70). The main reason for not showing more kinds of wearable devices is that these products are not yet mature and available (71). Therefore, the corresponding product display opportunities should be updated according to the development of the wearable device market in the future.

### 6.4. Future directions

Based on the conclusions of the above analysis, future research should extend this study in the following ways. First, by developing a wearable device-centered elderly medical market, the imbalance between the supply and demand of elderly care services caused by the unfair distribution of medical resources can be alleviated (72). The specific reason is that although current wearable medical devices can be used for health monitoring, most of them only act as superficial health stewards, which is far from the essence of intelligent medical care (73). However, the elderly have the greatest need for medical care (74); thus, there will be a market or a breaking point only by moving closer to the elderly medical market. Second, for elderly users in need, functional and portable wearable devices can be designed according to their own physical and psychological characteristics to fill the knowledge gap on wearable special equipment for elderly individuals. For instance, considering that many elderly people have poor vision and cannot operate wearable devices, the design of large screens and fonts can allow elderly people control the device as they want. Finally, for the relevant departments, it is recommended to incorporate wearable devices with special attributes for medical detection into the daily body monitoring of elderly individuals, thereby alleviating the shortage of national public medical resources. At the same time, relevant industry standards should be introduced to regulate industry operations, and legislation also needs to be passed to protect information and data.

## Data availability statement

The raw data supporting the conclusions of this article will be made available by the authors, without undue reservation.

## Ethics statement

Ethical review and approval was not required for the study on human participants in accordance with the local legislation and institutional requirements. The patients/participants provided their written informed consent to participate in this study.

## Author contributions

JC designed the study and wrote the manuscript. TW amended the manuscript and participated in the improvement of conceptual model and data processing. ZF reviewed the literature, revised the manuscript, and discussed the uncertainties with JC to reach consensus. HW gave many valuable suggestions for this manuscript. All authors have read and approved the final version of the manuscript for submission.
